# Rethinking disability: The need to rethink representation

**DOI:** 10.4102/ajod.v7i0.498

**Published:** 2018-05-10

**Authors:** Jenna-Lee Procter

**Affiliations:** 1 Department of Psychology, Stellenbosch University, South Africa

As a clinical psychologist working in the fields of intellectual disability (ID) and transgender mental health in South Africa, I found *Rethinking disability: World perspectives in culture and society* informative and refreshing. It has expanded on the social model by incorporating anthropological and sociological theories to offer a critical perspective of ableism, disability culture and the contextualisation of disability from a unifying disciplinary perspective.

There are two other book reviews which are easily accessible online (see Sharpe 2017 and Regler 2017), that provide excellent summaries and comments on *Rethinking Disability.* Rather than echoing many of their arguments in my review, I would like to offer an African take, as *Rethinking Disability* was submitted to the *African Journal of Disabilities* (AJOD) for review.

I was excited to review *Rethinking Disability* after thumbing through the introductory chapters and reading the back cover. I was intrigued by the concept of ‘disABILITY MUNDUS’, or:

world perspectives on disability that are contemporary in nature, in which we explore the contextualisation of disability in history, through the material and immaterial, its expressions in culture and society, its local and global nature, its educational context, and its trans – and post human contexts. (Devlieger et al. 2016:13)

This suggested that I would encounter various perspectives from people the world over who draw on critical theory and espouse principles of inclusivity, representation and intersectionality.

I was disappointed to discover, despite emphasising on disABILITY MUNDUS, that the volume’s dominant voice came from the white, global North. Only a few of the contributors were mixed race and none, as far as I could tell, were from Africa, Central or East Asia, India or Eastern Europe. I found this lack of representation problematic in a text that espouses ‘world perspectives’ and uses a title which implies that ‘world’ is synonymous with North America and Western Europe. The exclusion of African researchers from the global academy is indicative of the failure to decolonise, losing out on useful transnational and transferable identity capital. The majority of people with disabilities in fact live in the global South, and we have many competent, ardent and inspiring disability scholars and academics who could have contributed to *Rethinking Disability.*

By focusing on disability values in the United States, the introductory essay by Gary L. Albrecht set the tone for an essentially Western dominated anthology. Opening with an essay on ‘disability values, representation and realities’ in the African context, rather than with a Euro-American centric paper, would have sent a more meaningful message.

The local cannot meet the global when the majority world is predominantly studied and written about by people who do not hold citizenship here. Titling this anthology ‘world perspectives’, when it is written by people not actually from the places they write about, and who encapsulate experiences they have not lived, is problematic. Exceptions are Maha Damaj’s article on institutionalised children with visual impairment in Lebanon and the inclusion of Mimi Lusli in the essay on people affected by leprosy in Indonesia. Both articles on Africa were written by white Westerners. The essays are academically valuable texts … but politically? Much less so. The oppression of black voices is perpetuated when their realities and contexts are spoken about, explained and changed, and when black histories are still written up by the white man (Ndaba et al. 2017). Of course, that does not mean that white people cannot write about other cultures, the problem arises when other cultures are mostly being written about by white people. The essays in *Rethinking Disability* also adopted an academic tone, making it inaccessible to many readers whose first-, second- or even third-language is not English.

Most of the authors in *Rethinking Disability* have, in one way or another, called on the academy to rethink how disability is performed and represented. This is evident in terminology used: ‘misfitting’, ‘ghettoization’, ‘diseducation’, ‘double outcast’ and ‘conscientisation’. But the argument for inclusion of people with disabilities into academia must be crucially extended to race, gender, sexuality, class and nation.

The book had an opportunity to exemplify intersectional disability studies. The inclusion of queer identities, for example, would have been potentially meaningful. Many queer folx have disabilities; there is a higher rate of disability among gay, lesbian and bisexual adults when compared with heterosexual ones (Fredriksen-Goldsen & Barkhan 2012). The 2015 U.S. Transgender Survey revealed that 54% of trans people with disabilities said that they had attempted suicide (James et al. 2016). We cannot afford to exclude lesbian, gay, bisexual, transgender, intersex and queer (LGBTIQ) identities from disability studies. Yet, *Rethinking Disability’s* transmodernism does not apply to one of the most marginalised groups within disability.

Many parallels exist in the queer and disability rights movements as evinced in some of the Part 4 essays. Tanya Titchkosky’s discussion of the interrelation of bodies, environment and knowledge in a university setting highlights the exclusionary processes which transgender and gender-diverse people must navigate. The question of ‘to pee or not to pee?’, so aptly put, mirrors the lack of gender-neutral restrooms in academic institutions which apply methods of justification for excluding trans people similar to those applied to people with disabilities. Josephine Hoegaerts looks at the projects of normality and educational assimilation of children with hearing and speech impairments. Just as trans people are expected to conform to the gender binary, she spoke of the oralist approach through which children – to be ‘passable’ – were required to hide their deafness by becoming masterful lip-readers and skilful speakers.

The hegemony inherent in academia has excluded disabled, queer and black academics who could have contributed, literally, to *Rethinking Disabilities*. In South Africa, the #RhodesMustFall and #FeesMustFall movements are powerful acts of defiance by young, mostly black students in the struggle for university access. These movements challenge the status quo of white supremacy, privilege and colonisation (Ndaba et al. 2017), while thoughtful applications of intersectionality to disability studies affords us opportunities to be more self-reflexive and honest about our positions and privilege. In Africa, the need to bring race and queer issues to the fore in the contextualisation of disability remains. Unfortunately, *Rethinking Disabilities* has missed the opportunity to give prominence to this major gap in disability studies.

The essays written by those with disabilities themselves were really appreciated. ‘Nothing about us, without us’, a term made popular by James Charlton and originally coined by two South Africans, Michael Masutha and William Rowland, was championed in *Rethinking Disability* as approximately a quarter of the contributors are disabled. Their participation in academia can be understood as a political act, just as Jori de Coster spoke on how disabled people, in a theatre-setting in Kinshasa, DR Congo, are supplanting oppressive representation with ones that hold true to their own realities.

The two articles on ID by Michel Desjardins and Philip and Dianne Ferguson are invaluable contributions to the field of ID – an often overlooked area in disability studies. Both unfortunately focused on the perspectives of parents rather than those of PWID themselves. This raises issues around infantilisation (perpetuating the notion that PWID are dependent, eternal children) (Capri & Swartz 2018) and exclusion. Excluding PWID from participating in knowledge creation as co-researchers or even primary investigators has arguably been considered unethical and discriminatory (Capri & Coetzee 2012).

Judged separately and on its own merits, each article offers insightful, contextualised and critical accounts of disability studies. There is something for everyone in this collection of essays as the topics discussed span a wide array of interests and issues. In many ways, it serves as a reference book for scholars and academics. I wish that more consideration was given to decolonising academia and including more voices from the global South in a book on ‘world perspectives’. *Rethinking Disability* has achieved its goal of bringing ‘ableism inside to a place where the prosthesis is no longer the elephant in the room’; however, it has not managed to bring white, cishet privilege to a place where *#blacklivesmatter* and every other marginalised or underrepresented identity is no longer the elephant in the room.
